# Hand instrumentation provides improved tissue response over ultrasonic scaler and substantiates safe dental practice: An *in vivo* study in rats

**DOI:** 10.1371/journal.pone.0284497

**Published:** 2023-05-11

**Authors:** Juliano Milanezi de Almeida, Nathália Januario de Araujo, Henrique Rinaldi Matheus, Elisa Mara de Abreu Furquim, Bianca Rafaeli Piovezan, Luiz Guilherme Fiorin, Edilson Ervolino

**Affiliations:** 1 Department of Diagnosis and Surgery–Periodontics Division, School of Dentistry, São Paulo State University (Unesp), Araçatuba, Brazil; 2 Nucleus of Study and Research in Periodontology and Implantology (NEPPI), School of Dentistry, São Paulo State University (Unesp), Araçatuba, Brazil; 3 Department of Basic Science, School of Dentistry, São Paulo State University (Unesp), Araçatuba, Brazil; King Saud bin Abdulaziz University for Health Sciences, SAUDI ARABIA

## Abstract

**Objective:**

The aim of this study was to evaluate the effectiveness of hand debridement (HD) versus ultrasonic dental scaler (UDS) for the treatment of experimental periodontitis (EP) in rats.

**Material and methods:**

Thirty 3‐month‐old male rats were used. EP was induced around the mandibular first molars (right and left). Seven days after induction, the treatments with either HD (n = 30) or UDS (n = 30) were randomly performed in each molar. Euthanasia were performed at 7, 15, and 30 days after treatment. Histometric (percentage of bone in the furcation [PBF]), histopathological, and immunohistochemical (for detection of tartrate-resistant acid phosphatase [TRAP] and osteocalcin [OCN]). Parametric data (PBF and TRAP) was analyzed by One-way ANOVA followed by Tukey’s post-test. OCN was analyzed by Kruskal-Wallis followed by Student-Newman-Keuls post-test. The level of significance was 5%.

**Results:**

Group HD presented higher PBF and lower TRAP-immunolabeling at 30 days as compared with UDS in the same period (p≤0.05). Group HD presented higher OCN immunolabeling at 30 days as compared with 7 and 15 days (p≤0.05). Persistent and exacerbated inflammatory process was observed in some specimens from group UDS at 30 days, as well as the bone trabeculae presented irregular contour, surrounded by many active osteoclasts.

**Conclusion:**

Nonsurgical periodontal therapy with HD resulted in higher PBF and lower expression of TRAP as compared with UDS. Also, HD increased the expression of OCN over time.

## Introduction

Since the outbreak of the SARS-CoV-2 pandemic, saliva has been considered a non-negligible factor in its spread [1], therefore, dentistry was strongly affected due to the evident transmission paths in dental practice [2]. Aerosol transmission has aroused as one of the main concerns [2]. Although SARS-CoV-2 fostered attempts to reduce aerosol formation, especially when using ultrasonic scaling [3], aerosol management in dentistry ought to be considered critical regardless of the pandemic. The well-established impact of periodontal inflammation in the extraoral health [4] demands appropriate management of the periodontal status and accurate treatment of the periodontal diseases. The therapeutic success of the treatment of periodontitis relies on the removal of microbial deposits, both soft and hard, from the root surface [5]. Many discussions focused on the effectiveness of ultrasonic devices over hand instruments for the treatment of periodontitis [6]. Not only periodontal indices were assessed, but also the effectiveness of removing subgingival deposits and root irregularities.

The use of hand instruments is capable of removing surface irregularities that ease the accumulation of plaque and calculus, rendering contaminated roots free of detectable endotoxins [7]. On the other hand, techniques that lead to less removal of dental tissues, utilizing sonic and ultrasonic scalers, are also effective for the removal of plaque and calculus [8]. For long, reviews of various studies performed under different clinical scenarios attested a non-superiority relationship of hand versus mechanical instruments with regard to the removal of subgingival deposits [9–11].

Zhang et al. [12] performed a systematic review and meta-analysis of 8 full texts aiming to compare the effectiveness of non-surgical periodontal therapy with either ultrasonic or manual scaling at different initial probing pocket depths (PPD). Their concluding remarks highlighted that despite ultrasonic devices being efficient, manual scaling proved to be superior when initial PPD was 4–6 mm. In addition, the superiority of manual subgingival scaling over ultrasonic scalers was confirmed by the reductions in PPD and clinical attachment level (CAL), even when initial PPD was ≥6mm [12].

However, to the best of our knowledge, no studies assessed the histopathological, histometric, and bone-related biomarkers while comparing non-surgical periodontal treatment with hand instruments and ultrasonic scalers so far. These findings would be relevant to substantiate the biological paths by which hand instrumentation proved to be superior to ultrasonic scalers for the treatment of periodontitis [12]. Additionally, during the SARS-CoV-2 pandemic and others to come, this knowledge would be critical to halting the use of aerosol-forming devices (ultrasonic scalers) and, therefore, to reduce the contamination and dissemination of pathogens transmitted by the saliva [1, 13].

Hence, looking forward to identifying the microscopic tissue reactions in order to underline the safe dental practice during the SARS-CoV-2 pandemic, it is the aim of this study to assess the histopathological, histometric, and bone-markers related changes when comparing hand instrumentation and ultrasonic scalers.

## Materials and methods

### Animals and experimental groups

Thirty healthy 3‐month‐old male Wistar rats (*Rattus norvegicus albinus*), each weighing 200–250 g, were used in this study. The animals were housed in a temperature controlled environment (22°C±1°C, 70% humidity) with a 12-hour light-dark cycle and *ad libitum* access to water and food. The study followed a split-mouth, single blind, randomized, controlled design. It was conducted in accordance with the ARRIVE Guidelines: Animal Research: Reporting In Vivo Experiments [14], and approved by the Ethics Committee of animal use at the São Paulo State University under the protocol number 00905–2019 of São Paulo State University, UNESP, School of Dentistry, Araçatuba. Sample size was calculated based on experience and previous literature [15], in order to achieve a 0.8 power and 0.05 alpha error based on a 12% potential standard deviation, and the assumption that a 10% difference between groups/periods would be relevant. Histometric analysis was the primary outcome parameter used to determine sample size. The animals were allocated to the groups HD (hand debridement) and UDS (ultrasonic dental scaler):

- Group HD (n = 30): Experimental periodontitis (EP) induction and non-surgical treatment with scaling and root planing (SRP) by using manual curettes and subgingival irrigation with physiological saline solution (PSS);
- Group UDS (n = 30): EP induction and non-surgical treatment by using ultrasonic scaler and subgingival irrigation with PSS.

### Anaesthesia

The surgical procedures were stated by sedation and general anesthesia, obtained by the combination of xylazine hydrochloride (6 mg/kg of body weight) and ketamine hydrochloride (70 mg/kg of body weight) [16].

### Experimental periodontitis induction

To induce EP through polymicrobial colonization of the periodontium, each animal received a #24 cotton ligature in a submarginal position around the mandibular first molars (right and left) at day 0 [15].

### Non-surgical periodontal treatment

The non-surgical periodontal treatments were performed by the same calibrated examiner [JMA], 7 days after EP induction. All ligatures were removed prior to periodontal treatment. For each animal, the software Minitab® 17 (Minitab Inc., State College, PA, USA) was used to generate a randomization table for allocating each of the first mandibular molars to one of the treatments.

Group HD: standard mechanical treatment of SRP was performed with 13–14 mine five Gracey curettes (Hu-Friedy, Chicago, IL). The protocol adopted in this study was established by Almeida et al. [15], in which ten distal-mesial traction movements were performed in the buccal and lingual surfaces. Also, ten cervical-occlusal movements were performed in the distal and mesial surfaces.

Group UDS: P4-sensitive tips (Helse Ultrasonic, FL, USA) were used to perform ultrasonic scaling. Ten intermittent distal-mesial scanning movements were performed in the buccal and lingual surfaces, while cervical-occlusal movements were performed to reach the distal and mesial surfaces.

After the surgical interventions (induction and treatment), the animals were allowed to move freely. Morphine i.m. (2.5 mg/kg) was administered once every 24 hours during 3 days for analgesia.

### Euthanasia

**Fig 1:**
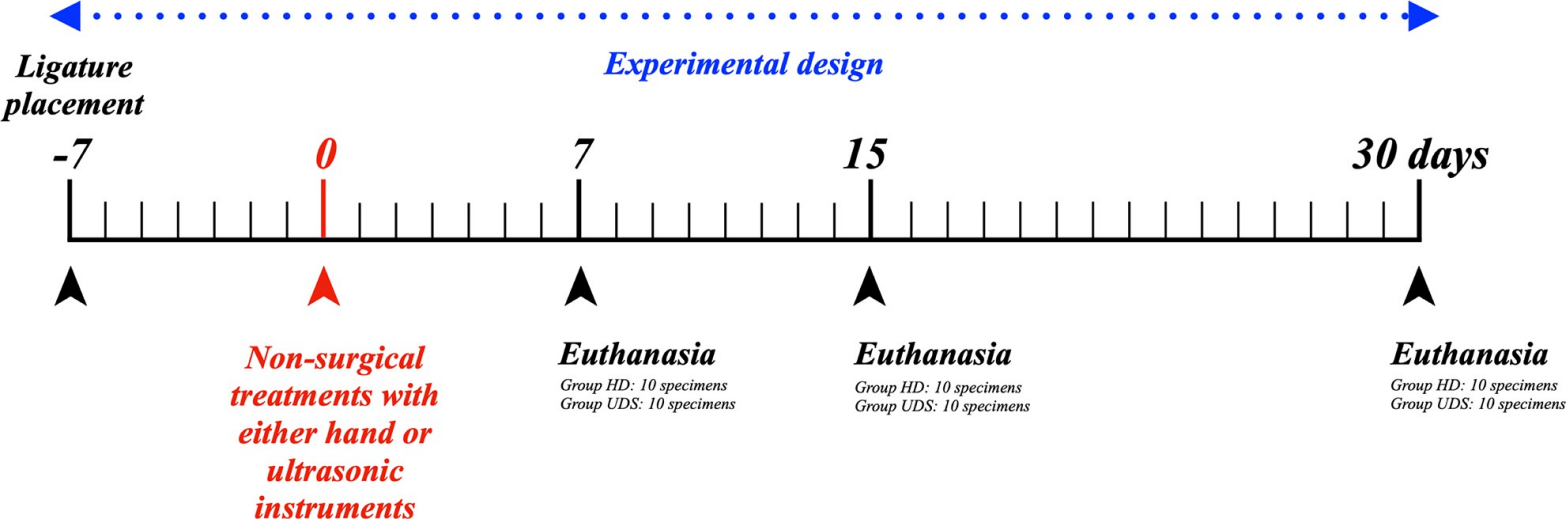
Scheme illustrating the procedures performed during the execution of the experiment for each group.

The euthanasia were performed at 07, 15, and 30 days after treatment, with a lethal dose (150 mg/kg) of sodium thiopental (Cristália Ltda., Itapira, SP, Brazil), consisting of 10 animals per group/period, thus totaling 20 specimens. The complete experimental design can be observed in Fig 1.

### Tissue processing

Conventional histological processing with paraffin embedding was performed for obtention of semi-serial 4 μm thick sections of the central portion of the furcation, from buccal to lingual progression. Five equidistant sections from each specimen were stained with Hematoxylin and eosin (H&E) for histopathological and histometric analyses of the percentage of bone in the furcation (PBF). Four additional sections from each specimen were subjected to the indirect immunoperoxidase method.

### Immunohistochemical processing

The indirect immunoperoxidase technique was performed in order to detect osteocalcin (OCN) and tartrate-resistant acid phosphatase (TRAP), following the protocols described by Matheus et al. (2021) [17]. The sections were divided into two batches, each of them incubated with the following primary antibodies: goat anti-osteocalcin (OCN) (anti-OCN, sc365797, Santa Cruz Biotechnology, Santa Cruz, CA, USA) and goat anti-tartrate-acid phosphatase (TRAP) (anti-TRAP, sc376875, Santa Cruz Biotechnology, Santa Cruz, CA, USA). Universal biotinylated secondary antibodies (anti-goat IgG + anti- rabbit IgG + anti-mouse IgG) (2 hr) and streptavidin conjugated with horseradish peroxidase (HRP) (1 hr) were used for signal amplification. The reaction was developed using the chromogen 3,3′-diaminobenzidine tetrahydrochloride (DAB chromogen Kit®; Dako Laboratories).

### Microscopy analysis procedure

The analyses were performed by examiners (NJA and EE) who were calibrated and masked to the treatments performed.

### Histometric analysis of the PBF

For histometry, five sections representing the center of the furcation were used. The software image J (National Institutes of Health, Bethesda, MD, USA) was used to conduct the histometric analysis of the PBF. First, the entire furcation area (FA) was delineated, measured in mm^2^ and considered 100% of the area to be analyzed. The area occupied by bone (BA) was delineated within the limits of FA, also in mm^2^. The ratio of BA to FA was calculated and the results were expressed as PBF. Remeasurement of the 4 sections stained with H&E was performed 1 week after the first measurement in order to assess the intra-examiner reliability and reproducibility.

### Histopathological analysis

The histopathological analysis was performed in the mesial, the furcation and the distal regions of the lower left first molars, by a masked histologist [EE]. It was conducted based on an adaptation of the criteria established by Gusman et al [18]. The parameters and scores are shown in Table 1.

**Table 1:** Parameters, scores and specimens’ distribution according to the histopathological analysis of the periodontal tissues in groups HD and UDS.

HISTOPATHOLOGICAL ANALYSIS										
PARAMETERS AND SCORES	PERCENTAGE OF SPECIMENS									
	EXPERIMENTAL GROUPS									
	HD							UDS		
	7 d	15 d			30 d			07 d	15 d	30 d
**INTENSITY OF THE LOCAL INFLAMMATORY RESPONSE**										
**(0)** absence of inflammation	-		-		40%			-	-	20%
**(1)** small amount of inflammatory cells	-		100%		60%			-	40%	80%
**(2)** moderate amount of inflammatory cells	80%		-		-			100%	60%	-
**(3)** large amount of moderate inflammatory cells	20%		-		-			-	-	-
**EXTENSION OF THE INFLAMMATION**										
**(0)** absence of inflammation	-		-		40%			-	-	-
**(1)**extending to part of the connective tissue	-		40%		60%			-	-	100%
**(2)** extending to the whole connective	100%		60%		-			100%	100%	-
**(3)** extending to the whole connective tissue and to the alveolar bone	-		-		-			-	-	-
**PATTERN OF THE CONNECTIVE TISSUE STRUCTURE**										
**(0)** moderate amount of fibroblasts and large amount of collagen fibers (dense connective tissue)	-		-			80%		-	-	-
**(1)** moderate amount of fibroblasts and collagen fibers	-		40%			20%		-	-	80%
**(2)** small amount of fibroblasts and collagen fibers	100%		60%			-		100%	100%	20%
**(3)** severe tissue breakdown and areas with necrosis	-		-			-		-	-	-
**PATTER OF ALVEOLAR BONE STRUCTURE**										
**(0)** bone trabeculae with regular contour, surrounded by many active osteoblasts, including areas of new bone formation	-			-			40%	-	-	-
**(1)** bone trabeculae with irregular contour, surrounded by many active osteoblasts and osteoclasts	20%			40%			60%	-	20%	100%
**(2)** bone trabeculae with irregular contour, surrounded by many active osteoclasts	80%			60%			-	100%	80%	-
**(3)** areas of necrotic bone and bone trabeculae with irregular contour, surrounded by many active osteoclasts	-			-			-	-	-	-

### Immunohistochemical analysis

The immunohistochemical analysis for both antigens was performed under light microscopy at × 400 magnification. The number of TRAP-immunoreactive (IR) cells with three or more nuclei was counted in the central area of the inter-radicular septum within an area of 1600 x 1200 μm. The coronary limit was the bone crest, which was spanned apically for a distance of 1200 μm. A semi-quantitative analysis of the immunolabeling of OCN was performed based on the criteria describe by Gusman et al [18].

### Statistical analysis

Data were analyzed using BioStat software (BioStat version 5.0, Belém, PA, Brazil). Cohen’s kappa coefficient was used to calculate the agreement between the measurements of PBF. The scores evaluated in the immunohistochemical analysis of OCN were submitted to analysis of variance with Kruskal–Wallis test and post-test of multiple comparisons of Student-Newman-Keuls. Parametric data (PBF and TRAP) were analyzed with analysis of variance (One-way ANOVA) and post-test of multiple comparisons of Tukey. The significance level was set as 5%.

## Results

Regardless of experimental group, all animals used in this experiment were healthy and no complications were observed during this study.

### Histometric analysis of the PBF

**Fig 2:**
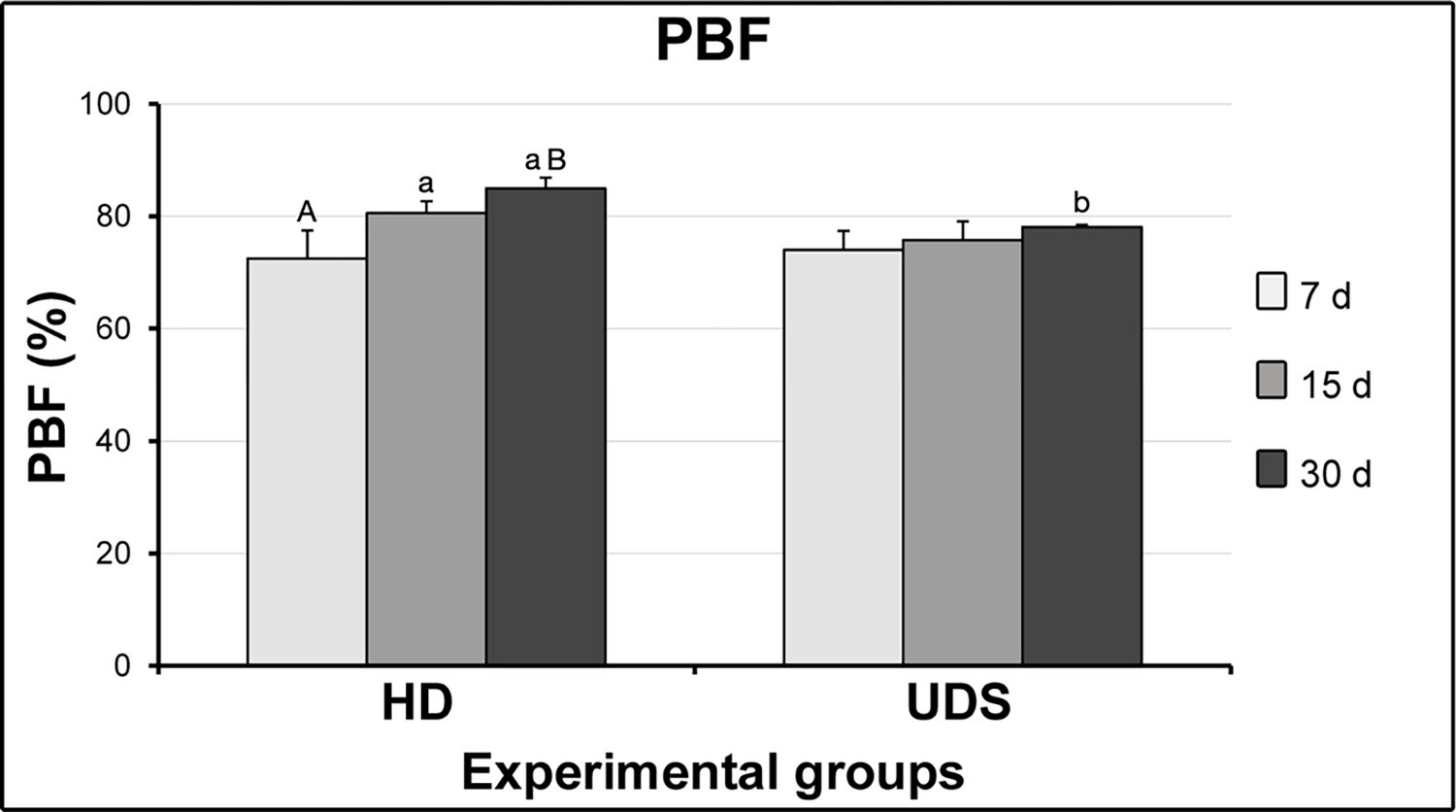
Graph showing the quantification (M±SD) of the percentage of bone in the furcation (PBF) for each group and period. Statistical tests: ANOVA and Tukey. The significance level was set as 5%. Different lowercase and capital letters in columns indicate that they differ from each other.

The agreement of 97.72% between measurements was identified through Cohen’s Kappa coefficient. Fig 2 shows the results of PBF for each group and period. The intragroup analysis of group HD presented higher PBF at 15 (p = 0.0000569) and 30 days (p = 0.0000080) as compared with 7 days. No intragroup differences were identified in group UDS (7 and 15 days, p = 0.2339; 7 and 30 days, p = 0.348; 15 and 30, p = 0.150). Higher PBF was identified in group HD at 30 days as compared with UDS at the same time point (p = 0.0009497) (S1 Dataset).

### Histopathological analysis of periodontal tissues

The histopathological analysis of the inflammatory parameters showed that the intensity of the inflammatory response decreased over time in both groups. However, while in all specimens from group UDS the inflammatory process extended through part of the connective tissue at 30 days, 40% of the animals in group HD presented complete absence of inflammatory infiltrate.

**Fig 3:**
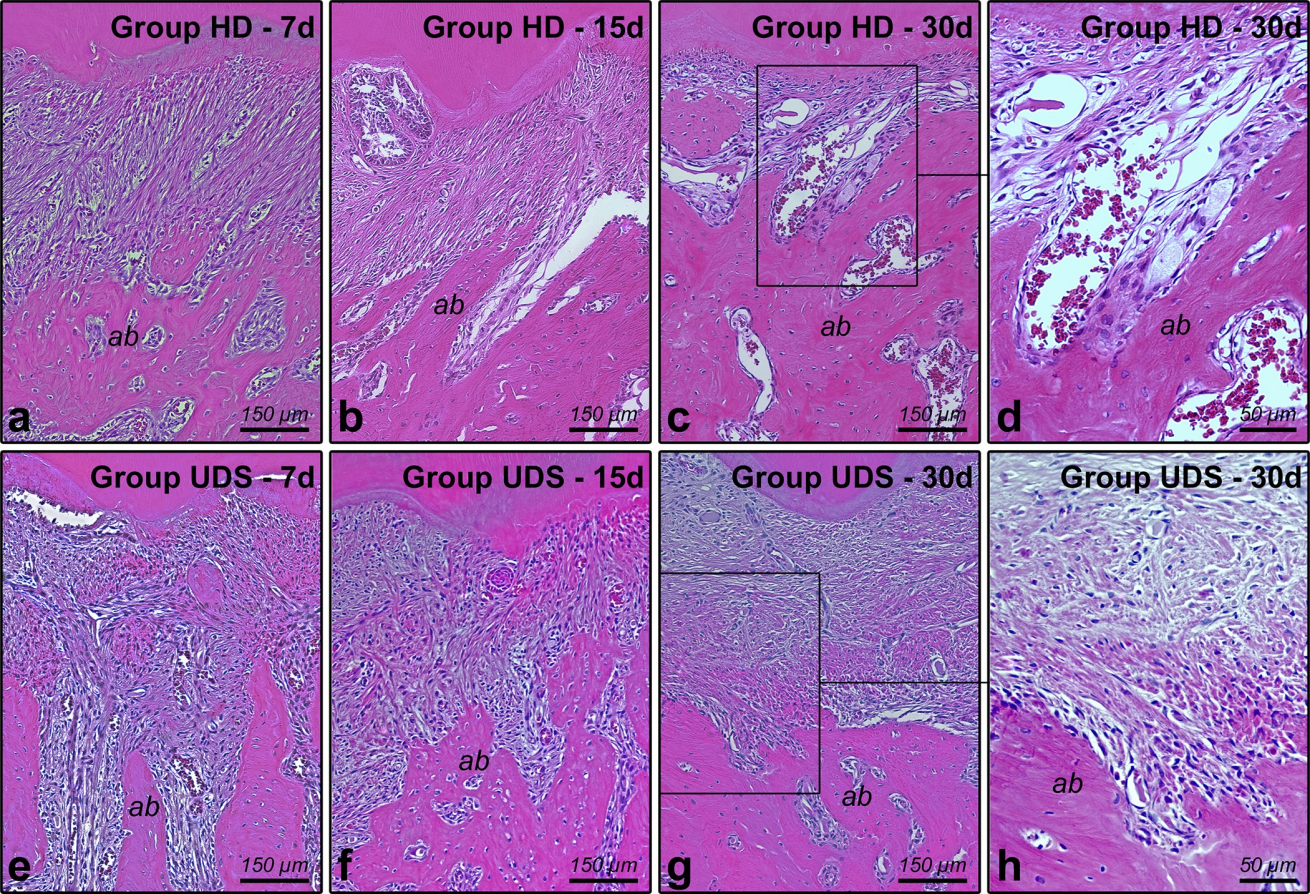
Histopathologic features of the periodontal tissues in the furcation region of the mandibular first molars. Abbreviations: ab, alveolar bone. A–H: photomicrographs showing the features of the periodontal tissues in HD 7d (A), HD 15d (B), HD 30d (C, D), UDS 7d (E), UDS 15d (F), and UDS 30d (G, H). Staining: Hematoxylin & Eosin. Scale Bars: A, B, C, E, F, ang G: 150 μm; D and H: 50 μm.

In group UDS, the pattern of the connective tissue structure presented either moderate or small amount of fibroblasts and collagen fibers at 30 days, while in HD, the connective tissue in most animals was composed of moderate amount of fibroblasts and large amount of collagen fibers (dense connective tissue) at the same time points. With regard to the pattern of alveolar bone structure, all specimens from group USD presented bone trabeculae with irregular contour, surrounded by many active osteoblasts and osteoclasts at 30 days, while in some specimens from group HD, bone trabeculae with regular contour were observed, surrounded by many active osteoblasts, including areas of new bone formation (Fig 3A through 3H). The parameters, scores, specimens’ distributions, and statistical analysis of periodontal tissues in HD and UDS are shown in Table 1.

### Immunohistochemical analyses

The high specificity of the indirect immunoperoxidase technique for detecting TRAP+-positive and OCN-positive cells was confirmed by the complete absence of immunolabeling in the negative controls. Were considered as immunoreactive cells (included for analysis) the ones presenting dark brown coloration, either confined to the cytosolic compartment (TRAP) or confined to the cytoplasm and poorly to the extracellular matrix (OCN).

**Fig 4:**
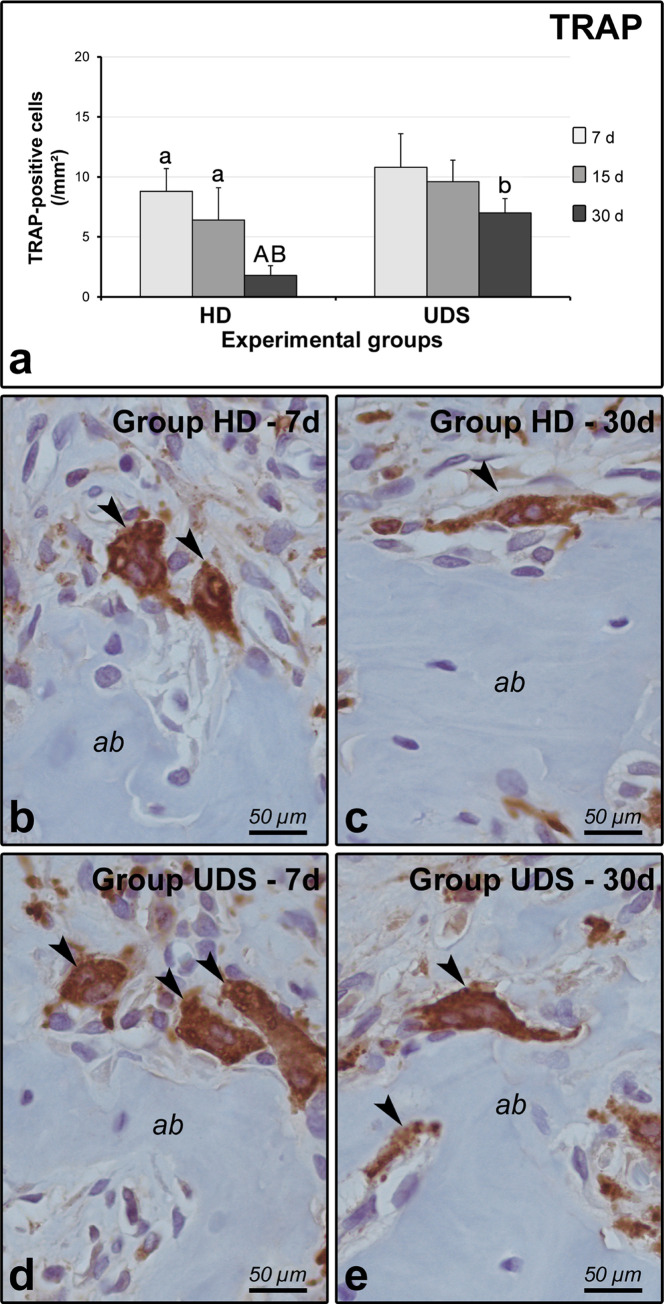
TRAP immunolabeling in the furcation region of the mandibular first molars. (A) Means and standard deviations (M±SD) of the number of TRAP-positive cells in the furcation for each group and period. Statistical tests: ANOVA and Tukey. The significance level was set as 5%. Different lowercase and capital letters in columns indicate that they differ from each other. B through E: photomicrographs showing the TRAP-positive cells (black arrowheads) in HD 7d (B), HD 30d (C), UDS 7d (D), and UDS 30d (E). Counter-staining: Harris’ hematoxylin. Scale bars: 50 μm.

The intragroup analysis in group HD showed lower number of cells immunoreactive to TRAP /mm^2^ at 30 days when compared with 7 (p = 0.0001493) and 15 days (p = 0.0013229). In intergroup analysis, group HD presented lower number of TRAP-positive cells/mm^2^ at 30 days when compared with UDS (p = 0.0001498) (Fig 4).

**Fig 5:**
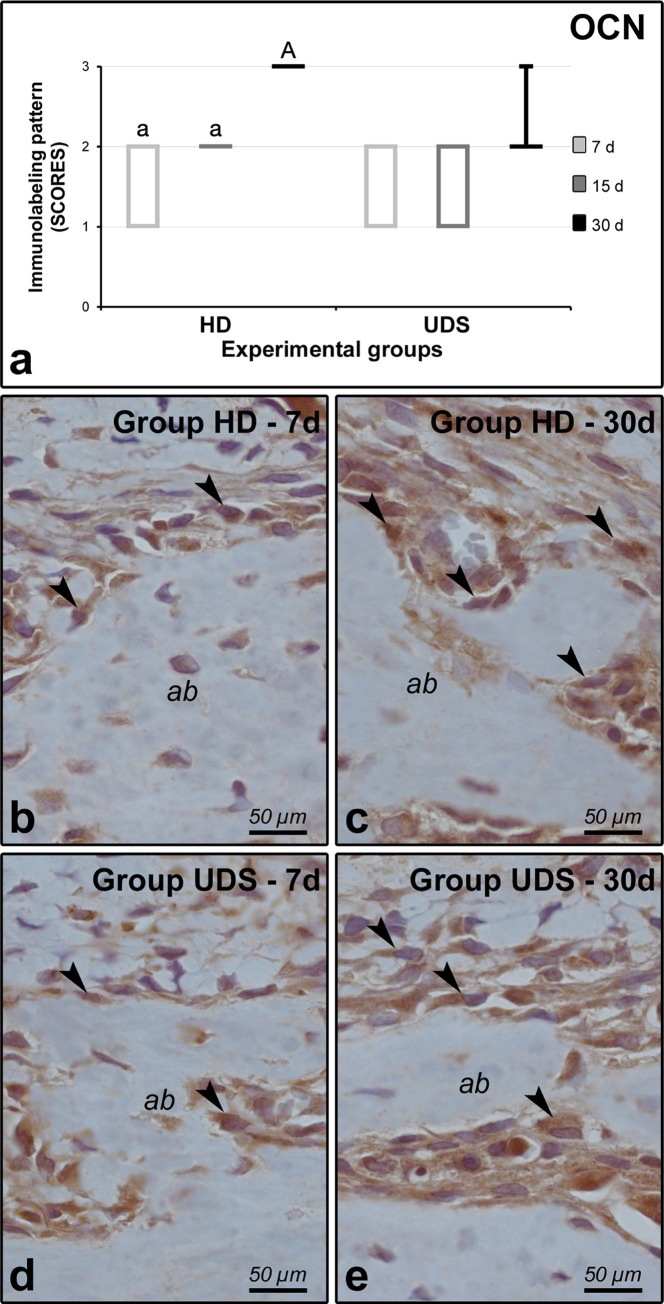
OCN immunolabeling in the furcation region of the mandibular first molars. (A) Graph showing the median and interquartile range of the scores for OCN in the furcation region for each group and period. Statistical tests: Kuskal-Wallis and Student-Newman-Keuls. The significance level was set as 5%. Different lowercase and capital letters indicate that they differ from each other. Symbols: †, statistically significant difference with 7 days in the same group (p≤0.05); ‡, statistically significant difference with 15 days in the same group (p≤0.05). B through E: photomicrographs showing the immunolabeling pattern of OCN in HD 7d (B), HD 30d (C), UDS 7d (D), and UDS 30d (E). Black arrowheads, immunolabeling cells. Counter-staining: Harris’ hematoxylin. Scale bars: 50 μm.

In intragroup analysis, group HD presented higher OCN immunolabeling at 30 days when compared with 7 (p = 0.0034) and 15 days (p = 0.0389) (Fig 5).

## Discussion

Non-surgical periodontal therapy stands for the most consolidated, secure, and effective form of treating periodontitis. Efforts have been put on this topic in order to determine which approach or combination (i.e. hand instrumentation and/or ultrasonic scaling) displays the most effective and predictable outcomes. Recently, the results of a systematic review and meta-analysis [12] indicated the superiority of hand instrumentation over ultrasonic scalers. This result plays an important role not only in periodontics, but also in the world’s public health during a pandemic as the SARS-CoV-2. It is known that aerosol forming devices consist of a critical transmission path in dental practice [2], and, hence, the biological superiority of manual curettes over USD could substantiate the conjunction of two overall strengths: 1- improved outcomes in periodontal therapy; and 2- controlled environment and safe dental practice during the SARS-CoV-2 pandemic. However, to the best of our knowledge, no studies have assessed, *in vivo*, the microscopic behavior of the periodontal tissues while comparing hand and ultrasonic debridement so far. The present experiment confirmed through histometric, histopathological, and immunohistochemical analyses, the superiority of SRP with manual curettes when compared with ultrasonic scaling for the treatment of experimental periodontitis in rats.

Animal experimentation is the first step for *in vivo* validation and essential for establishing cause and effect relationships. Despite the distinct metabolic rate, the rodent model of EP induction by ligature placement provides histopathologic features very similar to those observed in humans [19] and, also, evidence equivalent microbiota to the clinical scenario [20], thus allowing to identify the mechanisms enrolled with the hypothesis to be tested.

Effective tissue repair is critical for the survival of all living organisms [21]. Although the inflammatory response guides many aspects related to the successful conclusion of the repair process, it can become dysregulated or chronic, and this may lead to severe impairment to normal tissue architecture [22]. Hence, the persistent inflammation observed in group UDS at 30 days may have led to a higher degree of disruption of the connective and bone tissues’ structure. The final stage of tissue repair is characterized by the restoration of tissues homeostasis, identified by the phenomena of inflammatory cells leaving the site of injury or being eliminated through apoptosis [23]. Possibly, the effective removal of the bacterial colonies [24] and the smooth dental surface achieved with hand instruments provided a propitious environment for resolution of the repair process [25].

The concept of bone repair being represented as the mere imbalance between formation and resorption is no longer acceptable. It represents an osteoimmunological phenomenon characterized by coordinated overlapping phases [26]. The discovery of RANKL [27], a tumor necrosis factor (TNF) superfamily cell-surface cytokine responsible for activation, maturation, and survival of osteoclasts [28], begun to unravel the intricate interaction between the immune and skeletal systems. TNF-alpha directly enhances the expression of RANKL by osteocytes and promotes osteoclast formation, confirmed by the significant increase in the expression of TRAP [29]. Collectively, the persistent inflammation and the higher number of TRAP-positive cells in group UDS at 30 days evidenced the impaired reestablishment of the osteoimmunological homeostasis of the alveolar bone following periodontal treatment with an ultrasonic scaler.

The maintenance or recovery of the alveolar bone is the target of many periodontal therapies, especially in the furcation region [30]. In group HD, the optimal progression of the repair process over time culminated with a higher PBF at 30 days when compared with group UDS at the same time point. This result seems to be due to the halt on the progression of EP, restoration of tissues’ homeostasis, and increased expression of OCN at 30 days when compared with 7 and 15 days after treatment, in group HD.

The literature reports that both hand and ultrasonic scalers are effective in altering the subgingival microbiota to one compatible with periodontal health [31]. However, it is also reported that ultrasonic instruments leave behind a rougher surface [32]. The principal mechanisms considered as favoring the retention of microorganisms are selective adhesion and stagnation [33]. The roughness of intra-oral surfaces influences the initial bacterial adhesion as well as its stagnation. Additionally, an increase in surface roughness was found to result in faster colonization and faster maturation of the plaque, thereby increasing the risk of periodontal infections [34]. Therefore, substantiated by the literature, the persistence of periodontal inflammation in group UDS may not be the result of an inefficient removal of bacterial plaque by ultrasonic instruments. Instead, the surface roughness may have created an appropriate environment for faster recolonization, which supports the microscopic persistence of the inflammation over time in group UDS.

The positive rate of severe acute respiratory syndrome coronavirus 2 (SARS-CoV-2) in patients’ saliva can reach 91.7%, and saliva samples can also cultivate the live virus [35]. This suggests that the virus could be transmitted via saliva, which increases the risk of aerosol transmission in the dental office. Thus, SARS-CoV-2 has the potential to spread through droplets and aerosols in dental clinics. Ultrasonic instruments are responsible for massive aerosol formation during dental practice [4]. Recently, Kumar & Subramanian [36], while demystifying some mist upon the primary source of pathogens in dental aerosols, affirmed that the lack of accurate information impedes strong conclusions if the contamination in dental offices occurs either by the saliva or by the dental unit water. Therefore, saliva aerosol formation shall remain a concern for a safe dental practice.

Early data suggests that SARS-CoV-2 remains virulent in aerosol for hours and on surfaces for days [37]. Recently, a study performed by Allison et al. [38] demonstrated positive readings of dental aerosol at up to 2 m away from the source and, even though in low levels, ultrasonic scalers produced particles reaching up to 4 m. Alisson et al. [38] concluded that dental aerosol and splatter have the potential to be a cross-infection risk, possibly due to their wide reach (m) of the particles and the maintenance of SARS-CoV-2 viability and virulence on inanimate surfaces. No measure to deal with the transmission paths of SARS-CoV-2 or any other pathogens may compromise the quality of the treatment and its impact on patients’ health. As the present study didn’t provide microbiological data or quantitative assessment of the periodontal inflammation, the results and discussion presented in the present research shall be interpreted with caution. However, the better structure and cellularity pattern of the periodontal tissues, the reduced resorptive activity, and the increase of the bone mineralization/turnover marker (OCN), may support the superiority of hand debridement versus ultrasonic instrumentation, as well as substantiate the use of manual curettes for safe dental practice.

Within its limitations, this study indicates that HD results in higher PBF, lower expression of TRAP, and less intense inflammatory process as compared with UDS. Also, HD increased the expression of OCN over time.

## Supporting information

S1 Dataset PBF dataset. (XLSX)
